# Evaluation of Tunisian Olive Leaf Extracts to Reduce the Bioavailability of Acrylamide in Californian-Style Black Olives

**DOI:** 10.3390/antiox12010117

**Published:** 2023-01-03

**Authors:** Dalel Mechi, Francisco Pérez-Nevado, Ismael Montero-Fernández, Bechir Baccouri, Leila Abaza, Daniel Martín-Vertedor

**Affiliations:** 1 Laboratory of Olive Biotechnology, Centre of Biotechnology of Borj-Cedria (CBBC), Hammam-Lif 2050, Tunisia; 2 Faculty of Science of Bizerte, University of Carthage, Zarzouna 7021, Tunisia; 3 Área de Nutrición y Bromatología, Departamento de Producción Animal y Ciencia de los Alimentos, Escuela de Ingenierías Agrarias, Universidad de Extremadura, Avda. Adolfo Suárez s/n, 06007 Badajoz, Spain; 4 Technological Institute of Food and Agriculture (CICYTEX-INTAEX), Junta of Extremadura, Avda. Adolfo Suárez s/n, 06007 Badajoz, Spain

**Keywords:** Tunisia, olive leaf extract, antibacterial activity, gastrointestinal activity, acrylamide, phenolic compounds, table olive

## Abstract

The aim of this work was analyzing the use of olive leaf extracts (OLE) obtained from two local Tunisian olive tree cultivars ‘Chemlali’ and ’Sayali’ to reduce the acrylamide in Californian-style black olives. The phenol profile, antioxidant, and antibacterial activity of the two OLE extracts were evaluated. The principal phenols found were hydroxytyrosol (1809.6 ± 25.3 mg 100 g^−1^), oleuropein (2662.2 ± 38 mg 100 g^−1^) and luteolin-7-O-glucoside (438.4 ± 38 mg 100 g^−1^) presented higher levels in ‘Sayali’ variety. Small differences were observed between the two kinds of extracts used; the greatest activity of OLE was observed against *S. choleraesuis*, with values up to 50% inhibition. The extract of ‘Chemlali’ cultivar was added to the Californian-style table olive, improving its phenol content and its antioxidant characteristics without negatively affecting its sensorial characteristics; these olives showed the highest firmness and proper quality characteristics. The gastrointestinal activity on the acrylamide concentration showed a partial degradation of this compound through the digestion, although the addition of the extract does not seem influence in its gastrointestinal digestion. These findings prove the usefulness of by-products to generate a high-quality added-value product, and this would also be relevant as a step towards a more sustainable, circular economy model.

## 1. Introduction

The cultivation of the olive tree (*Olea europaea*) presents socioeconomic relevance in Mediterranean areas and the Middle East. In Europe, Spain exceeds 2.6 million hectares of olive groves, representing 21% of production worldwide [1]. This sector presents an important economic sector due to its capacity to generate employment in the Mediterranean Basin [2]. Mediterranean countries share similar climatic and agroecological conditions, since olive trees are often cultivated in shallow soils with low organic matter content, with values below 2% [3]. In Europe, responsible agricultural policies are currently being established for the cultivation of olive trees, which aim to be responsible in considering current challenges such as climate change and food needs with the forecast increase in the world population for the year 2050, as well as support policies for sustainable agriculture [4]. In Tunisia, the olive oil sector is one of the most important agri-food sectors since it contributes to the socioeconomic development of the country, being the most important olive-producing country in the Southern Mediterranean, being the fourth in production worldwide (172,000 t/year). After Spain, Italy, and Greece, with more than 35% of land devoted to olive cultivation [5]. In this country, several local olive cultivars are used; among the main olive tree cultivars in Tunisia are ‘Chemlali’ in the South and in the Center and ‘Chetoui’ in the North, representing more than the national production of olive oil [6]; other relevant cultivars are ‘Sayali’, ‘Neb jmal’, or ‘Tkobri’.

However, through the production of table olives and olive oil, these industries produce large amounts of residues, some of them, such as twigs or leaves, obtained during the pruning and olives harvesting [7,8] and, other, such as olive oil pomace, during the process of olive oil [9]. In recent years, studies to use these by-products have been made as a step towards a circular economy model, more sustainable, and, thus, meeting the Sustainable Development Goals of the United Nations [10]. Some recent studies have analyzed the use of olive leaf extracts during the fermentations of table olives to improve the nutritional value of the final product [11], to obtain a virgin olive oil enriched with aqueous extracts of olive leaf and olive oil pomace [12], to use the olive oil pomace as a source of high-added value compounds [9], or to produce chitosan-based-edible films containing Dried Olive Leaf Extract with antioxidant and antimicrobial activities [13]. Therefore, these by-products are considered of high added value to the food industry. Olive leaves have a complex composition, with different compounds, including polyphenols, triterpenic acids or sugars [14]. The main phenolic compound in olive leaves is oleuropein, but there are others, such as hydroxytyrosol, luteolin, apigenin, tyrosol, verbascoside, rutin, caffeic acid, chlorogenic acid, quercetin, and epicatechin, among others [14,15,16]. Due to its composition, a large number of health-promoting properties have been related with its antioxidant and antimicrobial activities and including its ability as modulator of the human immune response to protect against different diseases [17,18]. However, Medina et al. [7] indicated that the composition of bioactive compounds in olive leaves varies greatly among commercial samples.

There are several styles of preserving table olives, one of them is the Spanish-style processing of green olives, which consists of removing the bitterness with a treatment with NaOH solution, washing with water and preserving in brine for several months to occur fermentation process [14]. Another important olive preservation process is the Californian-style, used in the case of black olives, which consists of subjecting them to an immersion treatment in NaOH solution of variable concentration (1–4%) and in the presence of oxygen to facilitate the oxidation with subsequent washing. This process is repeated in several cycles to achieve the desired product [19]. Industrially, it is carried out in open tanks, whether made of concrete or polyester with inclined bottoms to facilitate the movement of the olives and their aeration. After the sodium hydroxide solution is removed, the olives are preserved in diluted brine or in water and later aerated to achieve their blackening [20]. During this kind of elaboration, a highly toxic compound, acrylamide, is produced due to the sterilization process. Different studies have been carried out to reduce the concentration of acrylamide in this product. Researchers have indicated that the strategy on lowering the F0 sterilization process is a well step to obtain a safe product with lower acrylamide concentrations [20,21,22]. Other studies used different additives, such as sodium bisulphite; amino acids (l-cysteine, l-arginine, proline, and sarcosine); calcium chloride; or natural extracts to control it [23,24,25]. In this sense, the addition of natural extracts based on olive leaves, with a high phenolic composition, has been studied in the last years to mitigate the acrylamide formation in table olives [26,27].

New studies would be of interest to the revalorization of food industry by-products located in the Mediterranean Basin. Thus, this work is focused on using two Tunisian local olive cultivars to obtain olive leaf extracts rich in bioactive compounds that could reduce the acrylamide concentration in Californian-style black olives. This could boost the Tunisian industry and, at the same time, improve the quality of oxidized olives.

## 2. Materials and Methods

### 2.1. Samples

The fresh olive leaf (1 kg) samplings of ‘Chemlali’ (E3) and ‘Sayali’ (E7) cultivars were carried out during the 2018/2019 crop year in Takelsa, Nabeul Region (Tunisia). Samples were washed with distilled water and dried at 25 °C in the Olive Biotechnology Laboratory (Tunisia). After that, olive leaves were dried at 50 °C until total moisture loss in an oven (model 210, J.P. Selecta^®^, Barcelona, Spain). Samples were crushed with a conventional mill until a particle size of 0.4 mm. They were stored until further use at −80 °C in bags (250 g) without air using a vacuum sealer (VS 100, Gustav Müller & Co. KG, Homburg, Germany).

Furthermore, Spanish table olives (*Olea europaea* L.) of the ‘Hojiblanca’ variety (20 kg) were harvested manually at green stage of maturation from an olive grove at “INTAEX-CICYTEX” Research Center (“Vegas Bajas del Guadiana” region, Badajoz, Spain). Samples were transported to the laboratory of CICYTEX to be processed as Californian-style black table olives.

### 2.2. Experimental Design of the Study

**Figure 1:**
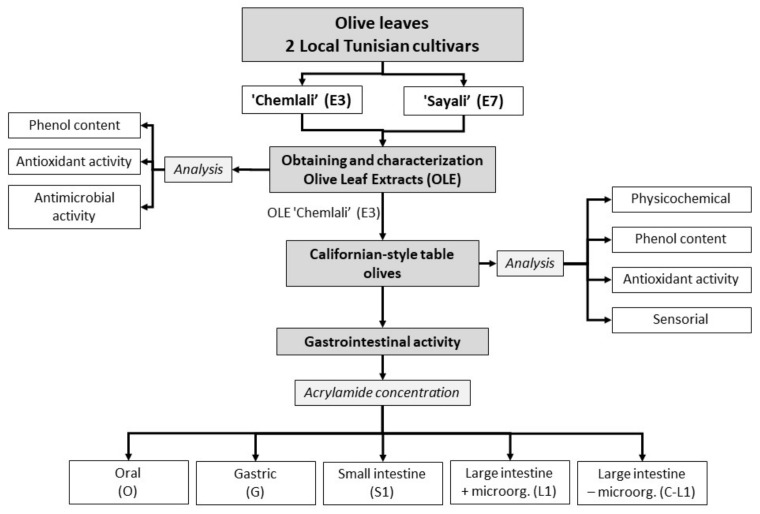
Experimental design of the study using olive leaf extracts (OLE) of local Tunisian cultivars.

The experiment design is displayed in the Figure 1. In this work, olive leaves obtained from two olive tree cultivars, ’Chemlali’ (E3) and ‘Sayali’ (E7), were used. Olive leaf extracts were obtained and characterized by analyzing their phenol content, antioxidant, and antimicrobial activities. After that, one of the extracts was selected and added to Californian table olives. Physicochemical and sensorial analyses were performed to the table olives, including phenol content and antioxidant activity. Finally, gastrointestinal activity of the table olives was carried on determining its effect on the acrylamide concentration.

### 2.3. Obtaining and Characterization of Olive Leaf Extract (OLE)

The olive leaves were extracted with water at 1:10 dilution at 60 °C for 1h using a kitchen robot following the method proposed by Martín-Vertedor et al. [14]. The final extract obtained was centrifuged to remove solid particles at 21,036× *g*. The filtered extract was kept refrigerated at 4 °C until analysis and after being added to Californian-style table olives.

#### 2.3.1. Determination of Phenolic Profile

The method of phenol extraction from the matrix, identification, and quantification of phenol profile was validated using the method proposed by [28]. For phenol extraction, 2 g of samples were extracted with 10 mL of methanol during 30 min in an ultrasonic bath (P-Selecta ultrasonic bath, mod 516, Barcelona, Spain). Prior the injection in HPLC equipment, the extracts were centrifuged at 1677× *g* at 4 °C during 10 min (Thermo Scientific Sorvall Legend XT/XF centrifuge, with a F13-14 × 50c carbon fiber rotor (Thermo Fischer Scientific, Waltham, MA, USA), and filtered through a 0.22 mm nylon syringe filter (FILTER-LAB, Barcelona, Spain). HPLC model Agilent 1100 (Agilent Technologies, Palo Alto, CA, USA) controlled by ChemStation for LC 3D with the Rev. B.03.02 system was used for the identification and quantification of phenolic compounds.

#### 2.3.2. Antioxidant Activity

The liquid extracts obtained for the phenol method were used to analyze the antioxidant activity using the method of extinguishing the absorption of the radical 1,1-diphenyl-2-picrylhydrazyl (DPPH reagent) [29]. Briefly, 2.7 mL of the methanolic solution of DPPH (20 mg L^−1^) was added to 300 µL of samples and mixed. The samples were incubated at room temperature in the dark for 1 h until readings were taken using a UV-3100 Spectrophotometer (Selecta, Barcelona, Spain) at 517 nm. The blank was made with methanol (J.P. Selecta, S.A). From the absorbances, the scavenging activity was calculated and hence they were compared on a calibration curve obtained for Trolox. The results were expressed in mmol Trolox kg^−1^ extract in Trolox equivalent antioxidant capacity (TEAC).

#### 2.3.3. Antimicrobial Activity

Antimicrobial activity was tested against a set of lactic acid and pathogenic bacteria (Table 1). Prior to the assay, lactic acid bacteria and pathogenic bacteria strains were cultured overnight at 37 °C in Man Rogosa Sharpe broth (MRS; Scharlab, Barcelona, Spain) and Brain heart infusion broth (BHI; Scharlab, Barcelona, Spain), respectively.

This assay was performed according to the modified method of Villalobos et al. [25] with an automated turbidometer Bioscreen C Analyzing System (Labsystems, Helsinki, Finland). Two media were used, MRS (for lactic acid bacteria) and BHI (for pathogenic bacteria), with 0.125% agar; the media was supplemented with three concentrations (1, 0.1, and 0.05%) of the two extracts (E3 and E7) and inoculated at 0.5% (*v*/*v*) from a suspension of tested microorganisms at 108 CFU mL^−1^. A positive control (containing inoculums but no extracts) and negative control (containing extracts but no inoculums) were included on each microplate. Samples prepared were added in 100-well microplates and were incubated at 37 °C for 70 h, and the optical density (600 nm) was measured at 15-min intervals. The assays were performed in triplicate. The inhibition (%) was calculated with the formula: Inhibition (%) = (OD_strain_ − OD_assay_) × 100/OD_strain_; OD_strain_ is the optical density of the growth of the test strain in the absence of extract, and the OD assay is the optical density of the growth of the test strain in the presence of the extract.

### 2.4. Californian-Style Black Table Olives Elaboration Process

Table olives harvested from the ‘Hojiblanca’ variety were processed as Californian-style black ripe olives [22]. For that, olives were stored in tanks of 20 L of capacity during 4 months with acetic acid solution (3% *v*/*v*) at room temperature. After that, olives were submitted to lye treatment (NaOH) with air oxidation until olives turn into black color. Olives were neutralized to pH = 7 with lactic acid (80% *p*/*v*), and finally, the black color was fixed with ferrous gluconate. They were washed to remove the excess of additive and olives were pitted and introduced into cans (150 g) with a new brine solution containing sodium chloride (3% *w*/*v*), ferrous gluconate (0.015% *w*/*v*), and 2 g L^−1^ CaCl_2_ (*w*/*v*).

### 2.5. Experimental Treatments of OLE Addition to Californian-Style Black Olives

Different experimental treatments were studied: (i) Californian-style black olives without any phenol addition in the govern liquid and (ii) oxidized black olive with OLE addition at 1:10 dilution (T). The govern liquid was neutralized at pH value of 7.2 by the addition of NaOH or HCl. All the cans were sterilized in an autoclave at 120 °C for 30 min. Cans were stored at room temperature before olives were analyzed. All the treatments were done in quintuplicate.

#### 2.5.1. Physicochemical Analysis

The physicochemical parameters were analyzed from Californian-style black olives [28]. TA.TX2 texturometer (Stable Microsystems, Godalming, UK) was used to assess the firmness of the olives. The results were expressed as the maximum force (kg). For the pH analysis, a Basic 20 pH meter (Crison Instruments, Barcelona, Spain) was used. Titratable acidity was determined with titration with sodium hydroxide (0.1 N) being expressed in grams of lactic acid 100 mL^−1^ of brine. Total chlorides were determined by titration with silver nitrate according to Mohr’s method using potassium dichromate as the indicator. The results were expressed as g 100 g^−1^.

#### 2.5.2. Sensory Analysis

The sensory attributes of Californian-style black olives evaluated were color, aspect, aroma intensity, bitterness, salty, acidity, hardness, defect, and global evaluation. These attributes were evaluated by a trained panel composed by eight trained tasters according to the standardized norm of the International Olive Council [30]. The tasters were all volunteers and signed an informed consent form agreeing to carry out the sensory analysis and allowing the use of the data provided. A sensory analysis was performed in triplicate.

#### 2.5.3. Gastrointestinal Activity of Table Olives with OLE Addition on Acrylamide Concentration

Table olives of ‘Hojiblanca’ cultivar with OLE addition were submitted to an in vitro gastrointestinal digestion according to the method proposed by Lodolini et al. [20]. Some (0.5 g) crushed olives were exposed to 1 mL of human saliva. The sample was mixed using an Orbital Shaker Incubator (Optic Ivymon System) for 10 s at 37 °C. The supernatant (Oral Digestion) was mixed to a 3.6 mL of simulated gastric fluid [14,31]. The mixture (Gastric digestion) was stirred with orbital shaking at pH 2.2 at 37 °C for 20 min. Subsequently, the sample was submitted to a small intestine, adding 3.6 mL of simulated intestinal fluid [14,31]. After, the mixture was shaken for 20 min at 37 °C to complete the intestinal digestion (Small intestine phase). Finally, Lactobacillus and Escherichia coli (105 CFU/mL) in a 50/50 ratio (*v*/*v*) were inoculated in the large intestinal phase. Then, the mix was incubated at 37 °C for 4 h (large intestine phase). A control without a microorganism addition was also performed (control large intestine phase). After each digestion step, the digestion mixtures were centrifuged at 21,036× *g* at 4 °C for 10 min and filtered through 0.22 µm nylon filters. The test was performed in quintuplicate.

Previously, during, and after the gastrointestinal digestion, the acrylamide concentration was determined in Californian-style table olives with and without OLE the ‘Chemlali’ E3 cultivar. The acrylamide determination in table olives was carried out in different stages of the gastrointestinal process: (i) oral (O), (ii) gastric (G), (iii) small intestine (SI), (iv) large intestine with microorganisms addition (LI), and (v) large intestine without microorganisms added (C-LI). Additionally, this analysis was made to table olives without digestion (Control).

Acrylamide determination was performed by the validated method according to Fernández et al. [31]. After acrylamide extraction with different cartridges, samples were analyzed using an Agilent 1290 Infinity II liquid chromatograph (Agilent Technologies), coupled with an Agilent 6460 triple quadruple mass spectrometer (Agilent Technologies).

### 2.6. Statistical Analysis

The statistical analysis was performed using IBM SPSS version 19.0 software for Windows (SPSS Inc., Chicago, IL, USA). Significant differences were established by analysis of variance (ANOVA). When the difference was between significant mean values, a test of comparison of means was performed using Tukey’s method (*p* < 0.05). Mean values and standard deviation are reported.

## 3. Results and Discussion

### 3.1. Characterization of OLE Obtained of Tunisian Olive Leaves

#### 3.1.1. Phenol and Antioxidant Activity of Tunisian OLE

Table 2 shows the profile of phenolic compounds for the olive leaf extracts of two varieties of Tunisian olives studied (‘Chemlali’, E3 and ‘Sayali’, E7). In total, 14 different phenols were identified and separated. It can be observed that there are significant differences in the concentrations of major phenolic compounds for the two varieties studied, standing out among the major ones are hydroxytyrosol (9278.0 ± 14.4 mg 100 g^−1^ and 4190.36 ± 5.70 mg 100 g^−1^) in oleuropein (15787.7 ± 84.7 mg 100 g^−1^ and 8485.60 ± 20.83 mg 100 g^−1^), quercetin (1120.8 ± 22.7 mg 100 g^−1^ y 2015.60 ± 210.10 mg 100 g^−1^), and luteolin-7-O-glu (2007.9 ± 7.3 mg 100 g^−1^ and 1855.35 ± 67.63 mg 100 g^−1^) in E7 and E3 extracts, respectively. However, there were no significant differences in the vanillic acid and gallic acid concentrations for the two varieties. The variation in the concentration of phenolic compounds between one variety and another is mainly influenced by geographical conditions, type of tree, and even age [19]. Hydroxytyrosol and tyrosol are two of the most representative simple phenolic compounds in olives and olive oil and are also highly appreciated industrially [21]. However, it can be observed that in the elaboration of this type of olive, the profile of phenolic compounds presents few identified compounds [21] in comparison with another type of fermentation style or with fresh nonfermented olives [22].

**Figure 2:**
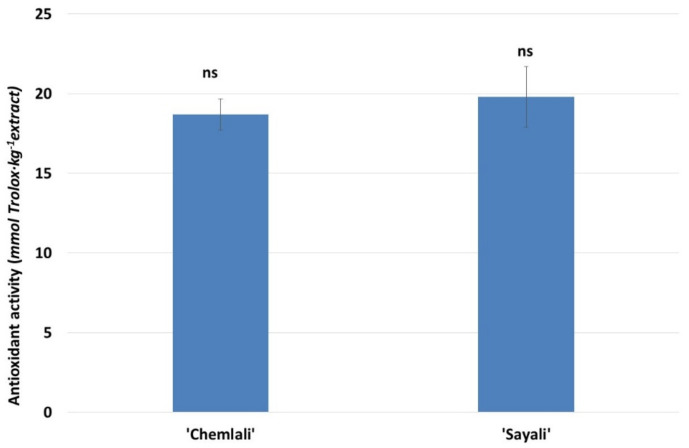
DPPH radical scavenging antioxidant activity (mg Trolox∙kg^−1^ extract) of ‘Chemlali’ (E3) and ‘Sayali’ (E7) olive varieties extracts. Different lowercase letters mean a statistically significant difference between the antioxidant activity for both varieties studied (Tukey’s test, *p* < 0.05). ns: not significant.

In Figure 2, the DPPH radical scavenging antioxidant activity antioxidant for the OLE of two varieties of Tunisian olives extracts was shown. No significant differences were found in the percentage of antioxidant activity for both varieties; however, the ‘Sayali’ (E7) variety presents a slightly higher percentage of antioxidant activity than the ‘Chemlali’ (E3) variety. The slight high antioxidant activity of ‘Sayali’ variety was confirmed with those obtained by high phenol profile of this variety. In other studies [8] analyzing the antioxidant activity of different extracts from varieties of olives grown in Tunisia such as ‘Chetoui’, ‘Meski’, ‘Oueslati’, and ‘Jarboui’, a high antioxidant activity with percentages higher than 70% was observed. Mechi et al. [23] also indicated a highly bioactive compound in OLE of local Tunisian varieties. The values obtained in this study are in line with other works found in the literature. In this sense, the antioxidant activity in OLE of ‘Arbequina’ [14] and adult ‘Chetoui’ [23] varieties presented values of 15.6 and 18.5 mmol Trolox kg^−1^ of extracts, respectively. These extracts and those found in this work was 15-fold higher than those obtained in other food such as virgin olive oil of the ‘Picual’ variety [12]. Finally, Baccouri et al. [24] indicated that OLE the ‘Oleaster’, ‘Chaaibi’, and ‘Zarrazi’ varieties presented antioxidant activity of 12, 10, and 8 mmol Trolox kg^−1^ of OLE, respectively.

#### 3.1.2. Antimicrobial Activity of Tunisian OLE

The antibacterial activities of the olive leaf extracts of ‘Chemlali’ (E3) and ‘Sayali’ (E7) cultivars were evaluated against three lactic acid bacteria and six foodborne pathogens including both Gram-negative and Gram-positive bacteria. In Table 3 is shown the percentage of inhibition of the different concentrations of these extracts against the bacteria assayed. The greatest inhibition activity was observed against *S. cholerasuis*, with values up to 50% inhibition at the three concentrations assayed with the two extracts, except for E3 0.05%. Other bacteria strains, such as *L. inocua*, *B. cereus*, and *S. aureus* were partially inhibited with some of the concentrations of the extracts assayed. *E. coli* and *E. faecalis* were the bacteria that represented higher resistance to the extracts. On the other hand, none of the three lactic acid bacteria (*L. sakei*, *L. curvatus*, and *P. acidilactici*) were inhibited by the two extracts assayed, to any of the concentrations tested. This could be considered as a positive point because the extracts assayed at these concentrations would not affect the olive fermentation [32].

When analyzing the differences in the effect of the two OLE assayed (‘Chemlali’ E3 and ‘Sayali’ E7), it was observed that both had similar antimicrobial activity, but their efficacy was different depending on the bacteria used and the concentration. E3 was more effective against *S. cholerasuis*, *E. coli*, and *E. faecalis*, whereas E7 was more effective against *B. cereus*, *S. aureus*, and *L. innocua*.

Although Gram-negative bacteria are assumed that are more resistant to antimicrobial compounds due to the composition of their outer membrane, in the present study differences between Gram-negative and Gram-positive bacteria were not found. Other studies [25] also found that a commercial olive leaf extract showed a great activity against several bacteria strains, mainly *Campylobacter jejuni*, *Helicobacter pylori,* and *S. aureus*, with a MIC lower than 1% (*v*/*v*). However, *Bacillus subtilis, E. coli*, and *Salmonella enterica* were included between the least susceptible bacteria. These authors indicated that olive leaf extract had not a broad spectrum of antimicrobial activity. Moreover, acid lactic bacteria such as *E. faecalis*, *Lactobacillus casei,* or *Lactobacillus acidophilus* were less affected by the extract [13] have proved the antimicrobial in vitro effect of films with Dried Olive Leaf Extract (DOLE) against bacterial strains (*Salmonella* and *Enterococcus*) in vitro, concluding that the effect was dose-dependent. The work of Gullón et al. [8] demonstrated the antibacterial properties of recovery of two extracts obtained from olive tree pruning and olive mill leaves against *E. coli*, *Salmonella* sp., *S. aureus,* or *L. innocua*, although with higher MIC than ours, ranging between 20 and 40 g L^−1^. There are other works performed with aqueous phenolic extracts obtained from plants and its antimicrobial effect. Villalobos et al. [25], using an extract from soy flour against foodborne pathogen, found a total inhibition of the pathogenic bacteria with 1.25 g L^−1^ of the extract. In the other studies [26] with different leaves extracts (lingonberry, bilberry, red currant, white currant, hawthorn, and chokeberry, among others), some of them showed the total inhibition of *S. aureus*, *B. cereus*, and *L. monocytogenes* but a scarce inhibition of *S. enterica* when applied doses of 20 µL in 300 µL of medium.

### 3.2. Influence of OLE Addition in Californian-Style Black Olives

The two varieties of Tunisian olives used in this work presented high concentrations of bioactive substance, but the ‘Chemlali’ variety was selected to be added as OLE in concentration 1:10 to the variety ‘Hojiblanca’ oxidized black in order to increase the concentration of phenolic compounds in order to figure out how bioactive compounds of the extract affected physicochemical parameters of table olives.

#### 3.2.1. Physicochemical Characteristics of Californian-Style Black Olives

The physicochemical analysis was performed on Californian-style black olives after the addition of OLE (Table 4). The firmness of table olives after OLE addition increased significantly. The higher phenol content could protect the olive cell wall for its degradation during the sterilization treatment [19,20,21,22]. In the same way, Caponio et al. [26] also indicated the high hardness of olives after being fermented in Spanish-style table olives. The inhibition of pathogens that degrade the cell wall by OLE could be the reason for this greater texture of the fruit. However, pH, acidity, and chlorides did not show statistical differences between treatments. These chemical parameters have been controlled artificially during the elaboration process of Californian-style table olives.

#### 3.2.2. Phenol and Antioxidant Activity of Californian-Style Black Olives

Table 5 shows the profile of phenolic compounds in the ‘Hojiblanca’ oxidized black olive used and the profile of phenolic compounds after the addition of OLE of the ‘Chemlali’ variety.

‘Hojiblanca’ olives present a profile of 11 phenolic compounds before adding olive leaf extract (OLE). This profile increases to 14 different compounds after adding OLE from the ‘Chemlali’ E3 variety, three of them coming from the OLE (quercetin, p-cumaric acid, and chlorogenic acid). An increase of 95.5% was observed in the total concentration of phenols in ‘Hojiblanca’ olives after the incorporation of OLE. The major compounds detected were: hydroxytyrosol, oleuropein, luteolin-7-O-glucoside, and quercetin 3-rutinoside and the minor ones vanillic acids and p-coumaric acid. The concentrations obtained of these compounds are in accordance with the literature, since the authors pointed out that OLE contain oleuropein as one of its main constituents in its phenolic composition, as well as other seroiridoids derived from the tyrosol structure [27]. Other major phenolic compounds present in OLE that have also been detected in this work are quercetin, luteolin-7-O-glucoside, and quercetin 3-rutinoside [28]. Due to the concentration of active phenolic compounds in OLE, its use in food has been reinforced by the European Food Safety Authorities, which grants them the declaration of health properties [33].

The percentage of antioxidant activity using the DPPH method for the ‘Hojiblanca’ olive produced in the Californian style without adding OLE and after the addition of OLE from the Tunisian ‘Chemlali’ variety (E3) is shown in Table 4. A notable increase in antioxidant activity occurs when OLE is added to olives made in the Californian-style. Other authors studying the antioxidant activity in OLE, also obtaining a high free radical scavenging capacity [24,25] with OLE presenting a high reducing power due to their high concentration of phenolic compounds and flavonoids [34].

#### 3.2.3. Sensory Characteristics of Californian-Style Black Olives

**Figure 3:**
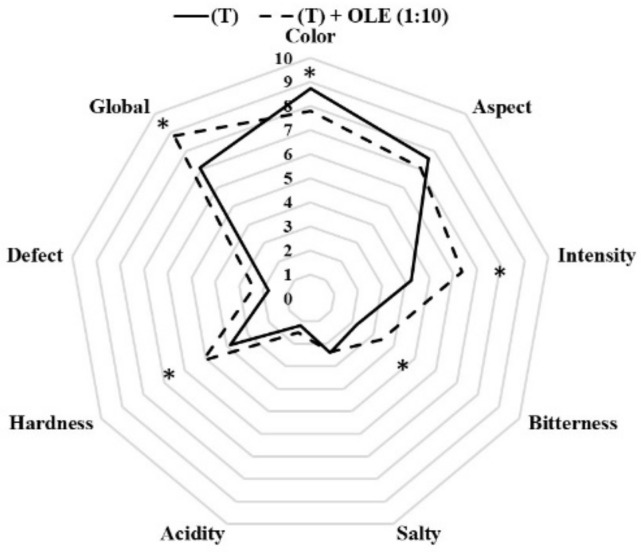
Spider plots of the sensory analysis of Californian-style black olives of the ‘Hojiblanca’ variety with (T + OLE) and without OLE addition (T). *: Statistically significant differences (Tukey’s Test, *p* < 0.05) among both treatments.

Figure 3 shows the Californian-style table olives sensory evaluation after OLE addition. The main differences in the evaluated attributes were shown in the color, flavor intensity, bitterness, hardness, and global evaluation of the final product. The attributes with the highest scores in Californian-style black olives with OLE addition were aromatic intensity, bitterness, hardness, and global evaluation, while the control olives (T) presented higher scores in the color and appearance of the final product. For the attribute Aromatic intensity, the values ranged from 4.2 to 6.4; for Bitterness, the values ranged from 2.2 to 3.4; for Hardness, it was rated between 3.8 and 5.2; and the Global evaluation for 7.1 to 8.8 points. Thus, panelist evaluated olives with the addition of OLE at a 1:10 concentration with the highest positive attributes. The herbaceous aroma of OLE could contribute to the highest evaluation of the olives and a good acceptance by tasters. Therefore, the addition of phenolic extracts to oxidized black olives contributes to a good strategy to increase the aromatic profile of these olives and to increase the phenolic profile of this type of olive. Different researchers [7,11,26] have evaluated the effect of OLE in the table olive sector, suggesting an increase in better texture and a positive overall evaluation of the final product. In the same way, Lalas et al. [35] showed that, although table olives with OLE addition increased slightly the bitter attribute, they presented equal overall acceptability and overall preference than olives without phenol addition.

#### 3.2.4. Effect of Gastrointestinal Activity on Acrylamide Content in Californian-Style Black Olives with OLE

**Figure 4:**
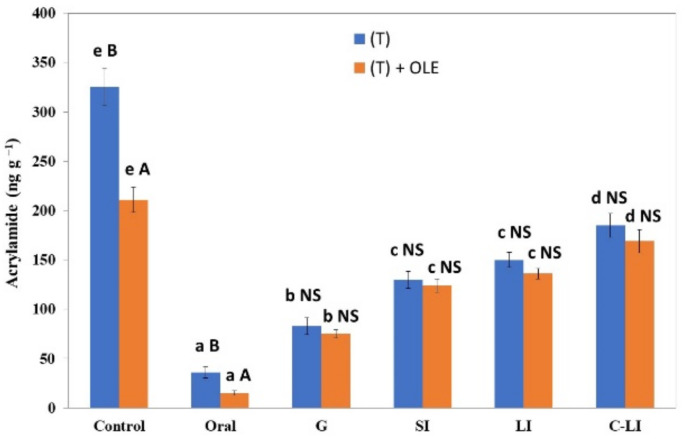
Gastrointestinal digestion of the acrylamide content (ng∙g^−1^) of ‘Hojiblanca’ table olives with (T + OLE) and without OLE E3 addition (T) in gastrointestinal stages (Oral, O; Gastric, G; Small intestine, SI; Large intestine without LI and with microorganisms C-LI) and without digestion (Control). Different small letters (a–e) indicate statistically significant differences (Tukey’s Test, *p* < 0.05) among the acrylamide content through the digestion phases, and upper letters (A,B) indicate statistically significant differences between olives submitted to OLE addition versus control.

In Figure 4 is shown the acrylamide concentration (ng g^−1^) after four gastrointestinal stages: oral (Oral), gastric (G), small (SI), and after large intestinal digestion with microorganisms (LI) and without them (C-LI), and a sample without gastrointestinal digestion treatment (Control) was also used. As we can observe, the concentration of acrylamide in the table olives was influenced by the presence of OLE; table olives with ‘Chemlali’ E3 OLE showed lower concentration of this toxic compound. However, during gastrointestinal digestion, no differences were found in the acrylamide concentration between the olives with and without the addition of OLE. If we compare acrylamide concentration in the control with the rest of the fractions, we can observe higher concentrations in the control samples. Thus, the gastrointestinal digestion could have produced a partial degradation of this compound. Additionally, the acrylamide concentration increased significantly during the digestion process; the lowest concentrations were found in the gastric stage. This could be due to the fact that the acrylamide contained in the olives was released during the digestion process as a consequence of enzymes, chemicals, and microorganisms; this is called the “matrix effect” by Lodolin et al. [20]. This effect is related to the ability of the olive pulp to protect against gastrointestinal digestion. González-Mulero et al. [36] proved the acrylamide–food matrix interactions; the presence of protein sources provokes a decrease in its bioaccessibility during and after in vitro gastrointestinal digestion. However, Sansano et al. [37], working with different food products (fries, crackers, biscuits, and other), found that, in general, the acrylamide content increased significantly after gastric digestion when compared with the initial food product.

Moreover, significant differences were found when compare the results obtained with and without microorganisms’ addition. The microorganisms seem to produce an interference in this liberation; they could produce a retention or even a partial degradation of this compound. These results were similar to those found by Fernández et al. [31]. These authors analyzed the effect of gastrointestinal process in vitro over the acrylamide concentration produced in four different table olives varieties. Although the acrylamide concentrations were dependent on the cultivar used [38], in general, the gastrointestinal process produced a release of this compound. Other authors have found similar results analyzing the effect of microorganisms associated to the digestion of other compounds such as phenols [14,39].

## 4. Conclusions

This work has shown how the use of waste from the olive industry, such as olive leaves, is an alternative for the incorporation of phenolic compounds in the food industry. OLE of two local olive cultivars ‘Chemlali’ and ‘Sayali’ have been shown to have a high concentration of phenolic compounds such as hydroxytyrosol, oleuropein, and luteolin-7-O-gluvoside. These compounds present in OLE inhibit the action of reactive species involved in oxidative damage, so they can be used in the food industry with positive benefits for health, as well as in the pharmaceutical and cosmetic industry. Additionally, the extracts were shown to have antimicrobial effects, this could be used in the table olive industry to increase the conservation of the product. When the extracts of ‘Chemlali’ were added to Californian-style table olives, a significant notable increase in the total concentration of phenols that produce an increase in its antioxidant activity. The sensory analysis showed that the OLE addition improved positive attributes in the final product and could be part of a strategy to increase the aromatic profile of these olives. Analyzing the effect of the gastrointestinal digestion on the acrylamide concentration, a partial degradation of this compound was observed, although an increase was observed during the digestion process due to the so called ‘matrix effect’. Additionally, the addition of microorganisms affects to the release of the acrylamide, producing its partial retention or degradation.

## Figures and Tables

**Table 1:** Bacteria strains used in the assay of antimicrobial activity.

Lactic Acid Bacteria	Pathogenic Bacteria
*Lactobacillus sakei* CECT5766	*Bacillus cereus* CECT 131
*Lactobacilus curvatus* CECT 904	*Escherichia coli* CECT 4267
*Pediococcus acidilactici* MS200	*Salmonella cholerasuis* CECT 4395
	*Staphylococcus aureus* CECT 59
	*Enterococcus faecalis* CECT 36
	*Listeria inocua* CECT 910

**Table 2:** Profile of phenolic compounds in the extracts of ‘Chemlali’ (E3) and ‘Sayali’ (E7) olive varieties. Different lowercase letters (a–k) mean a statistically significant difference between data in the same row, and different capital letters (A,B) mean significant differences between data in the same column (Tukey’s test, *p* < 0.05). N.S: not significant.

Variety		
	‘Chemlali’	‘Sayali’
Phenolic Profile (mg·100 g^−1^)		
Hydroxytyrosol	4190.4 ± 5.7 j A	9278.0 ± 14.4 i B
Tyrosol	177.6 ± 0.9 d A	258.3 ± 6.2 d B
PB1	198.1 ± 1.2 e NS	120.5 ± 7.9 c
Epicatechin	70.9 ± 6.5 c A	115.3 ± 0.0 c B
Verbascoside	652.7 ± 9.7 h A	782.7 ± 12.9 f B
Quercetin 3 rutinoside	558.5 ± 9.9 g A	709.4 ± 3.8 f B
Luteolin-7-O-glucoside	1855.4 ± 67.6 i A	2007.9 ± 7.3 h B
Oleuropein	8485.6 ± 20.8 k A	15,781.7 ± 84.7 j B
Quercetin	2015.6 ± 200.1 i B	1120.8 ± 22.7 g A
Gallic acid	2.1 ± 0.2 a NS	2.7 ± 0.2 a
Vanillic acid	0.6 ± 0.1 a NS	1.0 ± 0.1 a
Cafeic acid	257.4 ± 11.1 f A	311.3 ± 9.8 e B
p-Coumaric	1.2 ± 0.0 a NS	1.5 ± 0.0 a
Chlorogenic acid	45.8 ± 10.5 b A	60.4 ± 10.3 b B
Σ phenols	18,511.9 ± 344.3 a A	30,551.5 ± 180.3 b B

**Table 3:** Inhibition (%) of three concentrations of two olive leaf extracts obtained from ‘Chemlali’ (E3) and ‘Sayali’ (E7) cultivars, against different bacteria strains. Different lowercase letters (a–e) mean a statistically significant difference between data in the same row, and different capital letters (A–H) mean significant differences between data in the same column (Tukey’s test, *p* < 0.05).

Bacteria Strain	‘Chemlali’ (E3)			‘Sayali’ (E7)		
	1%	0.1%	0.05%	1%	0.1%	0.05%
**Lactic acid bacteria**						
*L. sakei* CECT5766	11.3 ± 1.6 dD	7.7 ± 1.5 cD	6.8 ± 1.2 bC	10.5 ± 2.5 dD	7.5 ± 1.1 cD	5.7 ± 1.1 aC
*L. curvatus* CECT 904	21.4 ± 3.5 eE	7.0 ± 3.9 cC	7.2 ± 3.6 cD	14.8 ± 3.9 dE	4.4 ± 2.7 aC	6.2 ± 2.4 bD
*P. acidilactici* MS200	10.0 ± 2.1 cC	0.52 ± 1.1 aA	0.4 ± 1.1 aA	8.8 ± 2.4 bC	0.2 ± 1.8 aA	0.8 ± 1.1 aA
**Pathogenic bacteria**						
*S. cholerasuis* CECT 4395	64.1 ± 3.0 eG	63.7 ± 2.8 dF	42.6 ± 3.7 aF	64.3 ± 2.7 eI	62.8 ± 2.3 cG	53.7 ± 2.7 bF
*E. coli* CECT 4267	2.8 ± 1.6 cA	0.8 ± 0.5 aA	0.3 ± 0.5 aA	1.2 ± 1.5 bB	1.7 ± 1.5 bB	0.4 ± 0.9 aA
*E. faecalis* CECT 36	9.3 ± 1.0 cB	0.3 ± 1.5 aA	0.6 ± 1.9 aA	0.3 ± 1.3 aA	1.4 ± 1.5 bB	0.7 ± 1.9 aA
*B. cereus* CECT 131	21.9 ± 2.4 cE	21.1 ± 1.4 cE	18.4 ± 1.5 bE	34.5 ± 2.8 dF	21.9 ± 1.4 cE	1.1 ± 2.7 aB
*S. aureus* CECT 59	29.9 ± 1.2 dF	3.6 ± 2.9 aB	4.6 ± 1.8 aB	41.7 ± 1.7 eG	20.4 ± 2.4 cE	9.3 ± 2.4 bE
*L. inocua* CECT 910	8.8 ± 3.5 bB	3.8 ± 2.2 aB	3.1 ± 2.7 aB	56.6 ± 2.9 cH	55.2 ± 3.5 cF	4.2 ± 2.0 aC

**Table 4:** Physicochemical parameters of Californian-style black olive of the ‘Hojiblanca’ variety with (T + OLE) and without the OLE E3 addition (T). Different lowercase letters (a,b) mean a statistically significant difference between data in the same row (Tukey’s test, *p* < 0.05). ns: not significant.

	Oxidazed Black Olive (T)	(T) + OLE (1:10)
Firmness (N)	3.7 ± 0.3 a	5.2 ± 0.4 b
pH	7.1 ± 0.1 ns	7.0 ± 0.1 ns
Acidity (g∙100 mL^−1^)	0.1 ± 0.0 ns	0.1 ± 0.0 ns
Chlorides (g∙100 g^−1^)	2.0 ± 0.1 ns	2.0 ± 0.1 ns

**Table 5:** Profile of phenolic compounds and antioxidant activity of ‘Hojiblanca’ oxidized black olives with (T + OLE) and without adding OLE 1:10 (T) of the ‘Chemlali’ cultivar E3. Different lowercase letters (a–b) mean a statistically significant difference between data in the same row, and different capital letters (A–K) mean significant differences between data in the same column (Tukey’s test, *p* < 0.05). nq: not quantified.

‘Hojiblanca’		
	Oxidazed Black Olive (T)	(T) + OLE (1:10)
Phenolic Profile (mg·100 g^−1^)		
Hydroxytyrosol	201.9 ± 26.9 a F	1809.6 ± 25.3 b I
Tyrosol	11.0 ± 3.0 a D	68.4 ± 7.4 b E
PB1	8.0 ± 2.9 a C	42.7 ± 3.6 b D
Epicatechin	1.2 ± 1.1 a A	35.6 ± 7.6 b D
Verbascoside	1.7 ± 0.3 a A	219.2 ± 0.3 b G
Quercetin 3 rutinoside	1.0 ± 0.8 a A	250.1 ± 10.0 b G
Luteolin-7-O-glucoside	1.1 ± 0.9 a A	438.4 ± 38.0 b H
Oleuropein	47.9 ± 9.7 a E	2662.2 ± 38.0 b J
Quercetin	nq	246.4 ± 27.1 G
Gallic acid	nq	2.2 ± 0.1 b
Vanillic acid	1.3 ± 0.2 a A	2.4 ± 0.4 b A
Cafeic acid	nq	96.1 ± 8.6 F
p-coumaric	3.6 ± 0.5 a B	5.7 ± 1.1 b B
Chlorogenic acid	nq	20.9 ± 3.0 C
Σ phenols	278.6 ± 4.5 a G	6186.1 ± 13.0 b K
DPPH radical scavenging activity		
DPPH (mmol Trolox·kg^−1^ extract)	0.8 ± 0.2 a	7.1 ± 1.5 b

## Data Availability

The authors confirm that the data supporting the findings of this study are available within the article, and the raw data that support the findings are available from the corresponding author upon reasonable request.
